# Development and validation of a new tool to measure the facilitators, barriers and preferences to exercise in people with osteoporosis

**DOI:** 10.1186/s12891-017-1914-5

**Published:** 2017-12-19

**Authors:** Isabel B. Rodrigues, Jonathan D. Adachi, Karen A. Beattie, Joy C. MacDermid

**Affiliations:** 1 McMaster University School of Rehabilitation Science, 1400 Main Street Wm, IAHS 308, Hamilton, ON L8S 4K1 Canada; 2 Department of Kinesiology, Waterloo University, 200 University Ave W, BMH 1100A/1102, Waterloo, ON N2L 3G1 Canada; 3 Department of Medicine, McMaster University, 25 Charlton Ave. E Room 501, Hamilton, ON L8N 1Y2 Canada; 4 St. Joseph’s Healthcare Hamilton, 25 Charlton Ave. E Room 501, Ontario, Hamilton L8N 1Y2 Canada; 5 Department of Medicine, McMaster University, 25 Charlton Ave. E Room 501, Hamilton, ON L8N 1Y2 Canada; 6 Hand and Upper Limb Center Clinical Research Lab, 930 Richmond, St. London, ON N6A 3J4 Canada

**Keywords:** Osteoporosis, Facilitators, Barriers, Exercise, Physical activity, Questionnaire, Checklist, Survey, Content validity, Instrument development

## Abstract

**Background:**

Despite the widely known benefits of exercise and physical activity, adherence rates to these activities are poor. Understanding exercise facilitators, barriers, and preferences may provide an opportunity to personalize exercise prescription and improve adherence. The purpose of this study was to develop the Personalized Exercise Questionnaire (PEQ) to identify these facilitators, barriers, and preferences to exercise in people with osteoporosis.

**Methods:**

This study comprises two phases, instrument design and judgmental evidence. A panel of 42 experts was used to validate the instrument through quantitative (content validity) and qualitative (cognitive interviewing) methods. Content Validity Index (CVI) is the most commonly used method to calculate content validity quantitatively. There are two kinds of CVI: Item-CVI (I-CVI) and Scale-level CVI (S-CVI).

**Results:**

Preliminary versions of this tool showed high content validity of individual items (I-CVI range: 0.50 to 1.00) and moderate to high overall content validity of the PEQ (S-CVI/UA = 0.63; S-CVI/Ave = 0.91). Through qualitative methods, items were improved until saturation was achieved. The tool consists of 6 domains and 38 questions. The 6 domains are: 1) support network; 2) access; 3) goals; 4) preferences; 5) feedback and tracking; and 6) barriers. There are 35 categorical questions and 3 open-ended items.

**Conclusions:**

Using an iterative approach, the development and evaluation of the PEQ demonstrated high item-content validity for assessing the facilitators, barriers, and preferences to exercise in people with osteoporosis. Upon further validation it is expected that this measure might be used to develop more client-centered exercise programs, and potentially improve adherence.

**Electronic supplementary material:**

The online version of this article (10.1186/s12891-017-1914-5) contains supplementary material, which is available to authorized users.

## Background

Osteoporosis is characterized by low bone mass and deterioration of bone tissue [1, 2]. The burden of this disease on individuals and the healthcare system is typically a result of fragility fractures that may result in immobility and hospitalization [3]. In 2010, it was estimated that 30% to 50% of women and 15% to 30% of men will suffer an osteoporotic fracture in their lifetime [4]. Osteoporotic fractures are more common than heart attack, stroke and breast cancer combined and hip fractures caused by this disease utilize more hospital bed days than diabetes, stroke, or heart attack [5]. As of 2010, the yearly cost to the Canadian healthcare system for treating an osteoporotic fracture was over 2.3 billion Canadian dollars [5]. Thus, fracture prevention strategies are key to reducing this burden. Exercise and physical activity is essential to preserve bone and physical function in patients with osteoporosis. A growing body of literature focuses on factors that affect exercise adherence including the facilitators and barriers to an exercise program.

Exercise as a means to prevent bone mineral density (BMD) loss has been explored extensively in the literature over the past two decades [2, 4, 6]. Exercise and physical activity are increasingly being recognized as a means to reduce the risk of osteoporotic fractures [2, 7] by increasing muscle mass and maintaining or increasing BMD [7–9]. Although the terms “exercise” and “physical activity” have distinct definitions, they are often used interchangeably in the literature. Physical activity is defined as “any bodily movement produced by skeletal muscles that result in energy expenditure” while exercise is “any form of physical activity that is planned, structured, repetitive and purposive” and used to maintain or improve physical endurance [10]. Since any form of activity is seen as beneficial to this population, this paper will not distinguish them.

A systematic review in 2013 indicated high variability in adherence to physical activity guidelines, with 2.4% to 83% of older adults meeting the recommendations [11]. This variation may indicate that a substantial proportion of people experience major barriers to exercise. In order to further outline the facilitators and barriers to exercise pertinent to patients with osteoporosis, we developed the Personalized Exercise Questionnaire (PEQ), to assess outcomes that were considered important by a panel of patients with low bone mass, physicians, therapists, and researchers. A comparable instrument that measures exercise beliefs exists; however this alone would not be sufficient to identify participant’s needs. The Exercise Benefits/Barriers Scale (EBBS) developed in 1987, has 43 questions and uses a 4-point Likert scale: *strongly agree, agree, disagree, strongly disagree* [12] and has a greater focus on attitudes and beliefs about exercise since the majority of items examine levels of knowledge about specific health benefits of physical activity. A study published in the British Geriatric Society Journal randomly selected 409 older adults determined that almost all participants (95%) believed physical activity was beneficial but barriers such as lack of interest, lack of transportation, pain, disliking going out alone, etc. were deterrents toward exercise adherence [13]. These barriers not covered in the EBBS may be more important determinants of exercise adherence. The EBBS also has minimal focus on the specific type of exercises that would be preferred and thus may not directly inform proper exercise prescription. Therefore, the PEQ was designed to address a different conceptual domain than the EEBS. The purpose of the PEQ is to collect information about self-reported facilitators, barriers, and preferences to exercise with the goal of supporting a better understanding of exercise adherence and patient centre exercise prescription for people with osteoporosis.

## Methods

The PEQ followed the two-step method described by Stein et al. [14] and Armstrong et al. [15], one involving instrument design and the other obtaining judgmental evidence. Instrument design was performed in a three-step procedure: A) content and domain specification; B) item generation; and C) instrument construction [16]. The second step, judgmental evidence (content validity) was conducted with a panel of experts [16].

### Step one: Instrument design

#### Content and Domain Specification & Item Generation

Items were generated from the literature retrieved from a PubMed and a CINHAL search to identify publications that evaluated exercise and/or physical activity in the osteoporosis population. Items were generated from a systematic review that evaluated the facilitators and barriers to exercise in patients with osteopenia or osteoporosis and a Belgian focus group study in older adults with osteoporosis that identified motivators and barriers to exercise [17, 18]. Due to limited research regarding specific motivators and barriers in the osteoporosis population, further items were attained from other populations: A) one from a Canadian focus group study that considered the facilitators and barriers to exercise in women aged 55–70 years [19], B) another from a study that evaluated exercise adherence in middle-aged adults [20], C) the third, from literature that evaluated the facilitators and barriers to exercise in community-dwelling adults [21], and D) the fourth, a literature review that identified barriers and facilitators to exercise in people with hip or knee osteoarthritis (OA) [22]. Women and older adults were chosen as similar populations to the osteoporosis group since this disease is more prevalent among women and is often diagnosed in older adults [5]. After extracting items and identifying duplicates, 37 unique questions were identified from the literature to construct a preliminary version of the PEQ.

Domains were selected using the Alternative Theory of Planned Behaviour, a combination of the Theory of Planned Behaviour and the Social Cognitive Theory [23]. This Alternative Theory analyzes four concepts: “perceived behavioural control,” “attitude toward exercising,” “environment,” and “normative beliefs” [23]. The 37 items were then categorized under one of the four theory concepts. Theory was originally used to create four domains for this tool; however, throughout the iterative development process, the titles were changed to reflect more patient friendly terminologies. For example “*normative beliefs*” was changed to “*support network*” and “*environmen*t” to “*access*”. Additional sections were added based on items found in the literature and some concepts from the Alternative Theory were combined to create new domains. Five domains were identified in the preliminary version: 1) my support network; 2) my access to exercise; 3) exercise goals; 4) my exercise preferences; and 5) my exercise barriers. For simplicity purposes, the following titles will be used when referring to a specific domain: 1) support network; 2) access; 3) goals; 4) preferences; and 5) barriers.

#### Instrument construction

Consistent with the recommendations from Stone, the preliminary version of this tool was circulated to an advisory committee for feedback [24]. A three-member committee was asked to evaluate the overall format, domains, and items of the questionnaire. The committee was comprised of a musculoskeletal researcher with a physiotherapy background, an investigator specialized in osteoporosis and exercise research, and a rheumatologist with a specialty in osteoporosis research. The questionnaire was revised through iterative feedback and submitted to a Delphi expert panel comprising of an osteoporosis researcher with a clinical degree in physiotherapy, two doctorate researchers specialized in osteoporosis research, and a kinesiologist. The Delphi technique, developed by the Rand Corporation, was used to seek convergence on this topic because it allows experts to work independently [25]. Each domain and item was reviewed for structure and clarity, redundant inquiries eliminated, and ambiguous wording modified.

### Step two: Judgmental evidence (content validity)

New surveys must be rigorously tested to ensure a tool is valid [26, 27]. Validity is defined as the extent to which any instrument measures what it is intended to [28]. For this reason, the development of the PEQ went through multiple iterations to ensure the survey was clearly worded, well defined, and covered topics important to patients with osteoporosis. Content validity measures how well items correspond or reflect a specific domain and are measured using quantitative techniques [27, 28]. Cognitive interview methods can explore how patients with osteoporosis might interpret the meaning of survey items [26]. Cognitive interviews were used to determine the following: 1) if participants understood the item; 2) if they understood the item the way the researcher intended, and 3) how participants calibrated the item and its response options. Lastly, focus groups were used to determine how respondents answer survey questions, identify potential problems that lead to response error, and comment on the overall format of the tool.

### Content validity

There are multiple methods for testing content validity. This study used one method that involved empirical techniques to calculate the index of content validity (CVI) and the content validity ratio (CVR) and semi-structure cognitive evaluations [15, 16]. The empirical techniques reviewed in this tool were:

1. *CVI:* CVI is the most widely reported approach for content validity in instrument development and can be computed using the Item-CVI (I-CVI) and the Scale-level-CVI (S-CVI) [16]. I-CVI is computed as the number of experts giving a rating of “*very relevant*” for each item divided by the total number of experts. Values range from 0 to 1 where I-CVI > 0.79, the item is relevant, between 0.70 and 0.79, the item needs revisions, and if the value is below 0.70 the item is eliminated [16]. Similarly, S-CVI is calculated using the number of items in a tool that have achieved a rating of “*very relevant*” [16]. There are two methods to calculating S-CVI, one is the Universal Agreement (UA) among experts (S-CVI/UA), and the second, the Average CVI (S-CVI/Ave), the latter being a less conservative method [16]. S-CVI/UA is calculated by adding all items with I-CVI equal to 1 divided by the total number of items, while S-CVI/Ave is calculated by taking the sum of the I-CVIs divided by the total number of items [16]. A S-CVI/UA ≥ 0.8 and a S-CVI/Ave ≥ 0.9 have excellent content validity [29].
2. *CVR:* The second type of empirical analysis was CVR, which measures the essentiality of an item [30]. CVR varies between 1 and −1, and a higher score indicates greater agreement among panel members [16]. The formula for the CVR is CVR = (Ne – N/2)/(N/2), where Ne is the number of panelists indicating an item as “essential” and N is the total number of panelists [16].

A cover letter and the PEQ were included with the content validity survey explaining why experts were invited to participate, along with clear and concise instructions on how to rate each item. To evaluate whether items were relevant, clear and essential, experts were given a critical appraisal sheet with the following four inquiries: 1) the relevance of each question in the tool (how important the question is); 2) the clarity of each question (how clear the wording is); 3) the essentiality of each question (how necessary the question is); and 4) recommendations for improvement of each question. The critical appraisal tool that experts used to rate the questionnaire is in Additional file 1: Appendix A. For the relevancy scale, a 4-point Likert scale was used and responses include: 1 = *not relevant,* 2 = *somewhat relevant*, 3 = *quite relevant,* and 4 = *very relevant.* Ratings of 1 and 2 are considered content invalid while ratings of 3 and 4 are considered content valid [31]. A 3-point Likert scale was used for the clarity and essentiality scale since answers can only be trichotomous. The clarity scale was: 1 = *not clear*, 2 *= item needs some revision*; and 3 = *very clear*, and for essentiality: 1 = *not essential*; 2 = *useful, but not essential*; and 3 = *essential* [15, 16]*.* Additional comments and recommendations by the experts were written on the hard copy of the questionnaire that was provided with the cover letter.

The recommended number of experts to review an instrument varies from 2 to 20 individuals [15]. At least 5 people are suggested to review the instrument to have sufficient control over chance agreement [16]. Content validity was determined using a number of experts (*n* = 6) that included an athletic therapist and a Ph.D. candidate from the University of Western Ontario, a physiotherapist, a chiropractor, and a family doctor from Toronto, Ontario and an orthopedic surgeon with a research background in osteoporosis from McMaster University. Experts were chosen based on the following guidelines: 1) worked in a medical or rehabilitation setting with patients with osteoporosis; or 2) published at least one article related to the care of patients with osteoporosis.

### Cognitive interviews

Cognitive interviewing is a methodology that examines how respondents comprehend, interpret, and answer survey questions [26]. The purpose of cognitive interviewing is to obtain information about the process respondents use to answer survey questions, identify potential problems that may lead to survey response error, and gain a better sense of their perception regarding items [32]. The question-and-answer model has been cited as a useful representation of how respondents answer survey questions [26]. This model suggests four interdependent elements, “comprehension of the information”, “retrieval from memory”, “decision processes”, and “response selection”, that interact together and predict how respondents make judgments about the level of detail needed to answer survey questions [33].

Cognitive testing was undertaken specifically with clinicians and patients to evaluate the cognitive process they followed to answer survey questions and to identify items that were not well understood. Techniques used to evaluate clinician and patient understanding of questions were a combination of both the think-aloud and verbal probing [33]. Together, these approaches were used to determine how well survey items were understood and how well different response options were reached. Specific think-aloud questions were: “please tell me what you are thinking as you answer this question” or “what steps are going through your head as you pick an option for this question” [32, 33]. Verbal probes were scripted or spontaneous and scripted questions included, “what do you think the question is asking you” and “please think aloud and tell me how you would answer this question” [26, 32, 33].

Cognitive interviews were done at McMaster University with 4 Ph.D. graduate students from the Department of Rehabilitation Science who had clinical backgrounds in occupational therapy, kinesiology, and physiotherapy. Interviews were also conducted with 2 patients from Hamilton, Ontario and 9 patients from London, Ontario and all patients had a diagnosis of osteopenia or osteoporosis. All interviews lasted between 1 to 1.5 h and were recorded and notes were taken. Analytic memos were created based on digital recordings and notes. Memos were coded into the following categories: 1) no problem with the item; 2) minor misunderstanding with the item; and 3) item unclear. Items marked “minor misunderstanding” were reworded, while those marked “unclear” were eliminated, reworded or integrated with another question.

### Focus groups

Focus groups are “informal discussions among selected individuals about a specific topic” [34] and can be used to follow up on issues revealed during cognitive interviews or used as a standalone protocol to generate ideas through group discussion [32]. Focus groups are typically more open-ended and less structured than cognitive interviews and can help elicit a greater range of responses. In this paper, focus groups were used to elicit respondents’ understanding, opinions, and views within the context of discussion and debate with other [34].

Two focus groups were held, one at McMaster University and the other at the University of Western Ontario, with 8 and 12 graduate students enrolled in the department of Rehabilitation Science and Physical Therapy, respectively. The majority of students were enrolled in a Ph.D. program. During the focus group, students were given a paper copy of the questionnaire and instructed to read each item and give their thoughts regarding the relevance, clarity and importance of each question. They were also asked to verbalize their thoughts about each item and whether it was in the correct domain. Although a digital recorder was not used, notes were taken during each focus group session.

## Results

The PEQ underwent 8 rounds of revisions from various expert groups. Table 1 summarizes major amendments in Additional file 2: Appendix B.

### Content validity results

All content validity (CVI and CVR) calculations were from the fifth version (6 domains, 35 questions) of the PEQ.

#### *I-CVI Results* (relevancy of individual items)

The I-CVI calculations for the relevancy of each item are in Table 2 (Additional file 2: Appendix B). Thirty-one items (89%) were marked as relevant and the I-CVIs ranged from 0.50 to 1.00. Twenty-two items had an I-CVI = 1.00, nine a score of 0.83, two a score of 0.67, and two a score of 0.50. The majority of items were considered relevant, with the exception of four questions: one on safety of the facility, one on support, and two about feedback.

#### *S-CVI Results* (relevancy of the overall questionnaire)

The S-CVI/UA = 0.63 and the S-CVI/Ave = 0.91. The *U*niversal *A*greement is calculated by adding all I-CVI’s equal to 1.00 (22 items) divided by 35, while the *Ave*rage takes the sum of all I-CVI (31.81) divided by 35. Overall, the Universal Agreement method demonstrates moderate content validity while the Average approach shows high content validity of the PEQ.

#### Kappa

Although CVI is extensively used to estimate content validity, Wynd et al. suggested that due to chance agreement this index does not consider the possibility of inflated values, and instead suggested a kappa statistic in addition to CVI be calculated [31]. Kappa provides the degree of agreement beyond chance, as is calculated using the following formula: K = (I-CVI – Pc)/ (1- Pc), where Pc = [N!/A!(N-A)!]* 0.5^N^ [16]. In this formula Pc = the probability of chance agreement; N = number of experts; and A = number of experts that agree the item is relevant. Kappa values above 0.74 are considered excellent, between 0.60 to 0.74 good and 0.40 to 0.59 fair. Kappa calculations are in Table 3 (Additional file 2: Appendix B).

#### CVR results

The CVR was generated for each item. Items that were marked not essential had a CVR < 0.99 (this value is based on the total number of experts, *N* = 6, and the numerical values of the Lawshe table) [35]. Nonessential items can be eliminated, but in this case were not. Twenty-two items out of 35 were marked not essential. Table 4 (Additional file 2: Appendix B) shows a sample of instrument items and the CVR calculations. Thirteen items had a CVR of 1.00, two a score of 0.67, seven a score of 0.66, four a score of 0.33, five a score of 0.00, three a score of – 0.33, and one a score of −0.66. The average CVR value was 0.53.

#### *Clarity Results* (individual items and overall questionnaire)

Clarity was calculated using 6 raters on a 3-point Likert Scale (1 = *not clear, 2 = somewhat clear, 3 = very clear*). Average clarity scores for individual items ranged from 2.33 to 3.00 with five items (14%) considered *very clear*. Five items had an average clarity score of 3.00, ten a score of 2.83, twelve a score of 2.67, three a score of 2.5, and five a score of 2.33. The overall clarity score of the fifth version of the PEQ was 2.69.

### Questionnaire refinement results

#### Version one and two (three-member panel)

There were two rounds of evaluations; in the first round, items were reworded for clarity and moved to more suitable domains. Two open-ended questions were added to include more information that may not have been captured through closed survey questions. These questions asked respondents to list up to three factors that would help them to exercise more often and up to three factors that prevented them from exercising more often. In the second round, one panel member suggested including several additional items regarding patient progression and feedback since patient perspective is important to be considered in an exercise program and enjoyment is both a predictor and an outcome of physical activity participation [36]. Members of the panel unanimously voted to include an additional domain regarding participant’s feedback and progress, so a sixth domain and 3 additional items were added. These three additional questions asked participants how they would like to receive feedback on their progression, the type of feedback they would like to receive and how often they would like to receive feedback. The third version of this questionnaire comprised 6 domains and 42 questions (40 categorical and 2 open-ended items).

#### Version three, four and five (Delphi panel and patients cognitive interviews from Hamilton)

The Delphi Panel underwent three rounds of refinement. In the first round, two questions on pain and one item on mobility were eliminated since the committee felt the items were adequately measured by other questions. One question regarding the type of exercise facility was moved from section two (access to an exercise facility) and combined with a question in section four (exercise preferences). In the second round, 2 out of the 39 items were removed because members felt respondents would not be able to answer questions about their DEXA and T-scores. One item regarding fractures was removed and turned into a sub-item question in section six. In the third round, two additional questions were added to section 5 about feedback and tracking. The panel thought it would be important to ask participants how they would like to track their exercise progress and if they would like to give feedback about the exercise program.

Patients diagnosed with osteopenia or osteoporosis also reviewed the fifth version of the questionnaire. Two patients from Hamilton, Ontario were recruited from the St. Joseph’s HealthCare’s Charlton Campus. One female with osteoporosis and one male with osteopenia suggested two additional questions for sections four and six, one on exercise times and the other on weather as a facilitator or barrier to exercise. The questionnaire was then updated to the sixth version (6 domains with 39 questions).

#### Version six (graduate student focus group + clinicians enrolled in the ph.D stream)

Two focus groups were held, one at McMaster University (*n* = 8) and the other at the University of Western Ontario (*n* = 12). Students at McMaster University were predominantly female (75%) and at the University of Western Ontario mostly male (66%). Both focus groups gave feedback on wording and terms consistency suggesting patients might confuse the terms “*exercise*” and “*workout*” or “*survey*” and “*questionnaire*”. It was recommended to use one term but not both. It was also suggested to use patient friendly terminology. Students suggested terms such as “*exercise movement*”, “*fracturing a bone*”, “*access to an exercise facility*” and “*limited range of movement*” be reworded. Students agreed that the font and spacing of sentences were adequate and the use of underlining was well done. They also believed all questions were appropriate and well worded with minor changes in terminology.

One-on-one interviews with clinicians in the Ph.D. stream (*n* = 4) were all females. All graduate students reviewed the sixth version of the questionnaire (6 domains with 37 questions) and were inquired to open an envelope and briefly look at each page of the survey. Subsequently, they were inquired to describe their initial impression and comment on the layout, response options, and overall flow of the questionnaire. Next, respondents were asked to go over each survey item and comment on its clarity, relevance, and importance. All participants stated the font, spacing, and length was acceptable however, specific item revisions were required. One proposed that the phrase, “if applicable, check any AIDS or DEVICES that you usual use…” should be changed to “if applicable, please check any mobility AIDS or DEVICES that you usual use.” Others suggested examples or additional words within parenthesis be added after items to clarify or define answer choices. For example in question 7, “*I have a safe place to exercise*”, the phrase “*e.g. proper space to exercise, dry and clean floors, good lighting, etc*.” was included in parenthesis after the question. They also suggested including, in parenthesis, after each question the following options: 1) “*check ALL that apply*”; or 2) “*check only one answer*” to clarify how many answers respondents could mark. Additional barriers to exercise were also suggested such as “*poor quality of sleep*” and “*not liking exercise*”. Items were added as sub-categorical answers to existing questions. One question regarding self-confidence was removed and turned into a sub-item question and another in section two about exercise safety was removed since most clinicians felt it was already measured by a previous question.

#### Version seven and eight (patient cognitive interviews)

Nine patients, five females and four males, from London, Ontario, were recruited from the Hand and Upper Limb Clinic (HULC) at St. Joseph’s Health Care Centre and all had a history of fractures. There were two rounds of judgment. Five patients assessed the seventh version of the questionnaire. Participants stated the instructions were simple and easy to understand and that items were clear but answer choices needed additional words or phrases. A few participants felt some questions were not applicable to them, and the inclusion of a “Not Applicable” category was important. Patients also found it difficult to complete section three, exercise goals. The questionnaire initially asked patients to rank the following goals in order of importance from 1 to 7, where 1 was the most important and 7 the least important. Answers were broad, with some patients ranking their goals from 1 to 7 and others ranking all goals with the same number. One participant thought he could only use the numbers 1 and 7 to rank. Section three was restructured to a 4-point Likert Scale (*not important, somewhat important, very important, not* applicable) and an open ended question that asked participants what their most important goal was, was added. The eighth version was then submitted for a second round of judgment. Four patients reviewed the eighth version of the questionnaire; there were no significant changes, with patients commenting on changes that were not applicable to the majority of people.

After compiling all advice from experts, the final version of the PEQ had 6 domains with 38 items, 35 close ended and 3 open-ended questions (see Additional file 3: Appendix C).

#### Readability grade levels

Readability levels were calculated for the eighth version of this questionnaire. The Flesch-Kincaid Grade Level and SMOG Index were calculated electronically (http://www.readabilityformulas.com/free-readability-formula-tests.php) and rate how easily sentences in the tool can be read and understood. The Flesch-Kincaid Grade level was 5.8 indicating it is easy to read and can be understood by an average 11-year old student. The SMOG Index was 6.9 demonstrating a seventh grade reading level.

## Discussion

This study developed and provided content validation of the Personalized Exercise Questionnaire (PEQ) that assesses multiple domains relating to the facilitators, barriers, and patient preferences in relation to exercise. Although the questionnaire was developed with the osteoporosis population as a primary target, the majority of items are not specifically related to osteoporosis suggesting that the questionnaire may be useful in a variety of other populations upon further validation.

The PEQ provides a unique self-report tool to assist with assessment of factors that may support or hinder the adoption and maintenance of regular exercise. Since it is well-known that adherence to exercise, physical activity, or home-based therapeutic exercise is problematic, it is the intention that the PEQ might support assessment of facilitators, barriers, and personal preferences in groups of people or be used to develop more personalized exercise recommendations for individuals to ultimately increase exercise participation. An article by Crombie et al. found the levels of knowledge about specific health benefits of exercise were high, yet the majority of older adults did not participate in any physical activity. The authors suggest national campaigns to encourage exercise and physical activity [13], however, persuading individuals with barriers to exercise may be difficult. Thus strategies to increase activity levels must include identifying the facilitators, barriers and patient preferences to an exercise program and compiling these factors using the PEQ to encourage participation.

The most common method for measuring content validity is calculating the Item-level CVI (I-CVI), however, an alternative, unacknowledged method to measure content validity is Scale-level CVI (S-CVI), which can be calculated using S-CVI/UA or S-CVI/Ave. The two approaches can lead to different values, making it difficult to draw the proper conclusion about content validity [37]. I-CVI measures the content validity of individual items while the S-CVI calculates the content validity of the overall scale. Most papers report the I-CVI or the S-CVI but not both. This paper considered both the I-CVI and the S-CVI since the S-CVI is an average score that can be skewed by outliers. The number of experts (*n* = 6) was considered adequate for content validation as the number of raters ranges from a minimum of 3 to a maximum of 10 [16, 30]. An I-CVI of 0.78 or higher is considered excellent. The I-CVIs of all items in the PEQ ranged from 0.50 to 1.00 with only four items having an I-CVI less than 0.78. This supports the conclusion that individual items were important and relevant to measuring the facilitators, barrier and patient preferences to an exercise program. The minimum acceptable S-CVI is considered to be any value between 0.80 to 0.90 [30, 37]. Two values were calculated: S-CVI/UA and S-CVI/Ave. The Universal Agreement approach suggested the overall content validity of the PEQ was moderate (S-CVI/UA = 0.63), while the Average method suggested high content validity (S-CVI/Ave = 0.91). Although the Universal Agreement method only considers items that have an I-CVI of 1.00 and may be considered more comprehensive than the Average approach, this method may be underestimating content validity of the overall questionnaire since the likelihood of achieving 100% agreement in all items decreases when the number of experts increases. The alternative and less constricted method is the S-CVI/Ave approach that may be overestimating content validity since the numerator in the Average technique will always be greater than the numerator of the Universal Agreement approach if I-CVI values are not all equal to 1.00. For this reason both the S-CVI/UA and the S-CVI/Ave were calculated and the true overall content validity of the PEQ may be somewhere in-between.

A less common way to calculate content validity is to use the CVR approach. This method determines how many raters mark an item as essential. Thirty-one items had a positive CVR value indicating at least half the raters considered the items to be essential, with an overall suboptimal content validity score, CVR = 0.53. It is possible that raters did not understand the item since only 14% of questions were considered *very clear.* Items were marked relevant indicating they were directly related to the topic but due to poor clarity raters may not have clearly understood what the item was measuring resulting in a low CVR score. The next step in instrument develop was to improve the item clarity using qualitative evaluations. Quantitative methods strongly supported individual items in the PEQ, and the use of cognitive interviews and focus groups were used to further refine the clarity of the language.

The complexity of doing numerous rounds of cognitive interviews and focus groups was to decide what information was relevant and when information was no longer considered important in tool development. The goal of cognitive assessments and subsequent revisions was to reach a point where there was sufficient evidence of no problems with item comprehension; at this point saturation has been achieved. Overall, the PEQ benefited from multiple consultations and iterative revision, which contributed to substantial changes in the types of items, concepts, wording, response options, and the overall structure of the questionnaire. Lack of clarity, misinterpretation, and ambiguity of items were the primary reasons for instrument modifications. It became clear that multiple iterations were essential since repeat consultations with people who had previously seen early versions had additional recommendations. There is no clear indication of the optimal number of revisions required to be certain that a measure is well developed [38]. However, the concept of saturation applies in iterative feedback when no recommendations being made are considered useful or that multiple respondents can agree upon. The PEQ underwent three additional rounds of revisions with a heterogeneous interview sample until feedback was not applicable to the majority. Content validity calculations (CVI and CVR) were not necessary to be measured again in the final version of the PEQ since content validity of individual items was excellent. Furthermore, rigorous qualitative research provided evidence of high content validity of the overall PEQ by reaching saturation through interviews with multiple experts.

Understanding factors affecting exercise adherence measured across multiple domains may help develop targeted interventions that may increase the quality and delivery of physical activity programs. This tool has potential applications in both the research setting and in clinical practice. Investigators can use this tool to survey their population of interest and use this information to inform decision-making about the type, frequency, and location of the exercise for the majority. The goal of designing an exercise program in research is to encourage individuals to continue the program long after the intervention has finished. Identifying an exercise program that increases muscle and bone mass, catered towards patient needs, will be one way of increasing exercise adherence. This tool can also help clinicians identify and design better exercise prescriptions for individual clients. It is important for healthcare providers to identify their patients’ facilitators, barriers, and exercise goals before giving specific recommendations since understanding these factors may result in better and more effective exercise prescriptions.

### Limitations

With any preliminary questionnaire there were some limitations to its design. The limitations of this study include: (1) potential lack of generalizability; (2) risk of using a self-reported measure; and (3) length of the questionnaire. Although the PEQ was designed for people with osteoporosis it may be applicable in elderly populations, but its generalizability to other clinical populations is unknown and must be tested. Secondly, with all self-reported measures there is a risk of recall bias or inflated answers to reflect lower impediments to exercise. The questionnaire also takes about 20 to 30 min to complete.

## Conclusion

The PEQ is the first instrument that assesses the facilitators, barriers and preferences to exercise in people with osteoporosis. The design of this questionnaire used a mixed-method approach to select items necessary to understand the facilitators, barriers, and preferences to exercise. The PEQ showed high content validity of individual items (I-CVI range: 0.50 to 1.00) and moderate to high content validity of the overall questionnaire (S-CVI/UA = 0.63; S-CVI/Ave = 0.91). Through qualitative methods, clarity of items was refined. Future physical activity or exercise interventions could benefit from using this tool to leveraging the facilitators and limiting the barriers to exercise to increase adherence to an exercise program.

## Additional files

Additional file 1: **Appendix A.** Critical appraisal of the checklist of facilitators and barriers to exercise. The content validity survey invited experts (healthcare professionals and researchers) to evaluate whether items in the PEQ were relevant, clear and essential. Additional file 1: Appendix A is a critical appraisal sheet with the following four inquiries: 1) the relevance of each question in the tool (how important the question is); 2) the clarity of each question (how clear the wording is); 3) the essentiality of each question (how necessary the question is); and 4) recommendations. (PDF 751 kb)

Additional file 2: **Appendix B.** Content validity results. Describes the 8 rounds of revisions. Table 1 summarizes the major amendments to the PEQ; Table 2 the I-CVI calculations; Table 3 kappa scores; Table 4 CVR values. Major revisions to the PEQ are also described in detail in this appendix. (DOCX 104 kb)

Additional file 3: **Appendix C.** The Personalized Exercise Questionnaire (PEQ). The PEQ was designed to identify possible facilitators and barriers to exercise in patients with osteopenia and osteoporosis. There are 6 domains (support network, access, goals, preferences, feedback and tracking, and barriers) and 38 questions. (PDF 121 kb)

## Abbreviations

BMD: Bone Mineral Density
CVI: Content Validity Index
CVR: Content Validity Ratio
EBBS: Exercise Benefits/Barriers Scale
I-CVI: Item Content Validity Index
OA: Osteoarthritis
PEQ: Personalized Exercise Questionnaire
S-CVI: Scale Level Content Validity Index
S-CVI/Ave: Scale Level Content Validity Index/Average
S-CVI/UA: Scale Level Content Validity Index/Universal Agreement
