# Supplementary material for: Development and validation of a new tool to measure the facilitators, barriers and preferences to exercise in people with osteoporosis
Source: BMC Musculoskelet Disord. 2017 Dec 19;18:540. doi: 10.1186/s12891-017-1914-5 (PMC5738121; doi:10.1186/s12891-017-1914-5)
Supplement: Supplementary file 1 — Appendix A. Critical appraisal of the checklist of facilitators and barriers to exercise. The content validity survey invited experts (healthcare professionals and researchers) to evaluate whether items in the PEQ were relevant, clear and essential. Additional file 1: Appendix A is a critical appraisal sheet with the following four inquiries: 1) the relevance of each question in the tool (how important the question is); 2) the clarity of each question (how clear the wording is); 3) the essentiality of each question (how necessary the question is); and 4) recommendations. (PDF 751 kb) [file 12891_2017_1914_MOESM1_ESM.pdf]

Critical appraisal of the checklist of facilitators and barriers to exercise

Title of Study: Reliability and validity study of the Facilitators and Barriers to Exercise Questionnaire

Student Investigator: Isabel B Rodrigues, B.Sc. (Hons), M.Sc. (candidate)
Department of Rehabilitation Sciences
McMaster University
Hamilton, ON, Canada
(905)-865-7426
E-mail: rodrigib@mcmaster.ca

WHAT IS THE PURPOSE OF THIS STUDY?

You are being invited to participate in a research study conducted by Isabel B Rodrigues to validate a new survey that will measure the facilitators and barriers to exercise in people with osteopenia and/or osteoporosis.

INSTRUCTIONS FOR VALIDATING THE QUESTIONNAIRE:

This tool has 35 questions that fall into the following six domains:

1. Community Support Network
2. Access to Community Facilities
3. Exercise Goals
4. Facilitators to Exercise
5. Feedback and Tracking
6. Barriers to Exercise

Kindly review this tool and provide your feedback on the following:

1. The relevance of each question in the tool (how important is the question)
2. The clarity of each question (how clear is the wording)
3. The essentiality of each question (how necessary is the question)
4. Recommendations for improvement of each question

If you have any comments or correction regarding the Facilitators and Barriers to Exercise Questionnaire, please make them on the copy provided.

Please complete the following questions:

1. Do you have a professional license?
   ☐ YES ☐ NO

   If YES, please specify:

   ____

2. For how long have you been practicing?

   ____

3. Where is your practice located (*city only)?*

   ____

Relevant Scale: 1= Not relevant; 2 = Somewhat relevant; 3 = Quite relevant; 4 = Very relevant

Clarity Scale: 1= Not clear; 2 = Item needs some revision; 3 = Very clear

Essential Scale: 1 = Not essential; 2 = Useful but not essential; 3 = Essential

**Section One**										
	How relevant is this item?				Is this item clear?			How essential is this item?		
**Q1.**	○1	○2	○3	○4	○1	○2	○3	○1	○2	○3
**Q2.**	○1	○2	○3	○4	○1	○2	○3	○1	○2	○3
**Q3.**	○1	○2	○3	○4	○1	○2	○3	○1	○2	○3
**Section Two**										
	How relevant is this item?				Is this item clear?			How essential is this item?		
**Q 4.**	○1	○2	○3	○4	○1	○2	○3	○1	○2	○3
**Q 5.**	○1	○2	○3	○4	○1	○2	○3	○1	○2	○3
**Q 6.**	○1	○2	○3	○4	○1	○2	○3	○1	○2	○3
**Q 7.**	○1	○2	○3	○4	○1	○2	○3	○1	○2	○3
**Q 8.**	○1	○2	○3	○4	○1	○2	○3	○1	○2	○3
**Section Three**										
	How relevant is this item?				Is this item clear?			How essential is this item?		
**Q 9.**	○1	○2	○3	○4	○1	○2	○3	○1	○2	○3
**Q10.**	○1	○2	○3	○4	○1	○2	○3	○1	○2	○3
**Q11.**	○1	○2	○3	○4	○1	○2	○3	○1	○2	○3
**Q12.**	○1	○2	○3	○4	○1	○2	○3	○1	○2	○3
**Q13.**	○1	○2	○3	○4	○1	○2	○3	○1	○2	○3
**Q14.**	○1	○2	○3	○4	○1	○2	○3	○1	○2	○3
**Q15.**	○1	○2	○3	○4	○1	○2	○3	○1	○2	○3
**Q16.**	○1	○2	○3	○4	○1	○2	○3	○1	○2	○3
**Section Four**										
	How relevant is this item?				Is this item clear?			How essential is this item?		
**Q17.**	○1	○2	○3	○4	○1	○2	○3	○1	○2	○3
**Q18.**	○1	○2	○3	○4	○1	○2	○3	○1	○2	○3
**Q19.**	○1	○2	○3	○4	○1	○2	○3	○1	○2	○3
**Q20.**	○1	○2	○3	○4	○1	○2	○3	○1	○2	○3
**Q21.**	○1	○2	○3	○4	○1	○2	○3	○1	○2	○3
**Section Five**										
	How relevant is this item?				Is this item clear?			How essential is this item?		
**Q22**	○1	○2	○3	○4	○1	○2	○3	○1	○2	○3
**Q23**	○1	○2	○3	○4	○1	○2	○3	○1	○2	○3
**Q24**	○1	○2	○3	○4	○1	○2	○3	○1	○2	○3
**Q25**	○1	○2	○3	○4	○1	○2	○3	○1	○2	○3
**Q26**	○1	○2	○3	○4	○1	○2	○3	○1	○2	○3
**Section Six**										
	How relevant is this item?				Is this item clear?			How essential is this item?		
**Q27**	○1	○2	○3	○4	○1	○2	○3	○1	○2	○3
**Q28**	○1	○2	○3	○4	○1	○2	○3	○1	○2	○3
**Q29**	○1	○2	○3	○4	○1	○2	○3	○1	○2	○3
**Q30**	○1	○2	○3	○4	○1	○2	○3	○1	○2	○3
**Q31**	○1	○2	○3	○4	○1	○2	○3	○1	○2	○3
**Q32**	○1	○2	○3	○4	○1	○2	○3	○1	○2	○3
**Q33**	○1	○2	○3	○4	○1	○2	○3	○1	○2	○3
**Q34**	○1	○2	○3	○4	○1	○2	○3	○1	○2	○3
**Q35**	○1	○2	○3	○4	○1	○2	○3	○1	○2	○3
