# Supplementary material for: Development and validation of a new tool to measure the facilitators, barriers and preferences to exercise in people with osteoporosis
Source: BMC Musculoskelet Disord. 2017 Dec 19;18:540. doi: 10.1186/s12891-017-1914-5 (PMC5738121; doi:10.1186/s12891-017-1914-5)
Supplement: Supplementary file 2 — Appendix B. Content validity results. Describes the 8 rounds of revisions. Table 1 summarizes the major amendments to the PEQ; Table 2 the I-CVI calculations; Table 3 kappa scores; Table 4 CVR values. Major revisions to the PEQ are also described in detail in this appendix. (DOCX 104 kb) [file 12891_2017_1914_MOESM2_ESM.docx]

**Appendix B**

**Table 1: Iterative Summary of changes to the PEQ**

| **Version Number** | **Reviewers** | **Total Number of Items in the beginning/ Total Number of Items in the end** | **Substantial (important) changes** |
| --- | --- | --- | --- |
| Version 1 | Three-member panel | 37 / 39 | - Items rearranged to the most appropriate domains - Addition of 2 open ended questions (patients to list up to 3 facilitators and barriers to exercise) |
| Version 2 | Three-member panel | 39 / 42 | - Addition of 1 domain (Section Five: “Feedback and Tracking”) - Addition of 3 questions on “feedback and tracking” |
| Version 3 | Delphi panel of experts | 42 /38 | - Removal of 4 questions (2 on pain, 1 on mobility, and 1 about the exercise facility) |
| Version 4 | Delphi panel of experts | 38 /35 | - Removal of 2 questions (1 on a patient’s last DEXA scan and 1 on a patient’s T-score) - Removal of 1 barrier question on fractures (turned into a sub-item question) |
| Version 5 | Delphi panel of experts  Patients from Hamilton, Ontario | 35 / 39 | - Addition of 2 questions in the “Feedback and Tracking” - Addition of 2 questions (1 on weather, 1 on exercise times) |
| Version 6 | Clinicians enrolled in the Ph.D. Stream  Two student focus groups | 39 / 37 | - Removal of 1 question on confidence (turned into a sub-item question) - Removal of 1 question in Section 2 about exercise safety (question already measured by another item) |
| Version 7 | Patients from London, Ontario | 37/38 | - Revised response of Section 3 on “Exercise Goals” - Addition of 1 open ended questions regarding “the most important goal” in Section 3 - Change from a 3 point to a 4-point Likert scale for sections 1, 2 and 3 to add a *Not Applicable* box |
| Version 8 | Patients from London, Ontario | 38/38 | - No significant changes made; changes applied to a limited number of individuals |

**Table 2: Calculation of the I-CVI for relevancy and clarity for each item (version 5)**

| **Item** | **I-CVI (Relevancy)** | **Interpretation** | **I-CVI (Clarity)** | **Interpretation** |
| --- | --- | --- | --- | --- |
| Q1 | 1.00 | Relevant | 0.33 | Not Clear |
| Q2 | 1.00 | Relevant | 0.83 | Clear |
| Q3 | 1.00 | Relevant | 0.83 | Clear |
| Q4 | 1.00 | Relevant | 0.83 | Clear |
| Q5 | 1.00 | Relevant | 0.67 | Not Clear |
| Q6 | 0.50 | Eliminated | 0.67 | Not Clear |
| Q7 | 0.50 | Eliminated | 0.33 | Not Clear |
| Q8 | 1.00 | Relevant | 0.83 | Clear |
| Q9 | 1.00 | Relevant | 1.00 | Clear |
| Q10 | 1.00 | Relevant | 0.83 | Clear |
| Q11 | 0.83 | Relevant | 0.67 | Not Clear |
| Q12 | 1.00 | Relevant | 1.00 | Clear |
| Q13 | 1.00 | Relevant | 1.00 | Clear |
| Q14 | 1.00 | Relevant | 1.00 | Clear |
| Q15 | 1.00 | Relevant | 1.00 | Clear |
| Q16 | 0.83 | Relevant | 0.67 | Not Clear |
| Q17 | 1.00 | Relevant | 0.67 | Not Clear |
| Q18 | 1.00 | Relevant | 0.83 | Clear |
| Q19 | 0.83 | Relevant | 0.83 | Clear |
| Q20 | 0.83 | Relevant | 0.67 | Not Clear |
| Q21 | 0.83 | Relevant | 0.67 | Not Clear |
| Q22 | 1.00 | Relevant | 0.83 | Clear |
| Q23 | 0.67 | Eliminated | 0.67 | Not Clear |
| Q24 | 0.83 | Relevant | 0.83 | Clear |
| Q25 | 0.67 | Eliminated | 0.83 | Clear |
| Q26 | 1.00 | Relevant | 0.67 | Not Clear |
| Q27 | 1.00 | Relevant | 0.67 | Not Clear |
| Q28 | 1.00 | Relevant | 0.83 | Clear |
| Q29 | 0.83 | Relevant | 0.50 | Not Clear |
| Q30 | 1.00 | Relevant | 0.67 | Not Clear |
| Q31 | 1.00 | Relevant | 0.50 | Not Clear |
| Q32 | 1.00 | Relevant | 0.83 | Clear |
| Q33 | 0.83 | Relevant | 0.67 | Not Clear |
| Q34 | 0.83 | Relevant | 0.67 | Not Clear |
| Q35 | 1.00 | Relevant | 0.67 | Not Clear |

NOTE: Number of items considered relevant and clear by all experts, N = 6.

**Table 3: Kappa Score for relevancy of each item (version 5)**

| **Item** | **Pc (Probability of chance agreement)** | **Kappa statistic** | **Interpretation** |
| --- | --- | --- | --- |
| Q1 | 0.016 | 1.00 | Excellent |
| Q2 | 0.016 | 1.00 | Excellent |
| Q3 | 0.016 | 1.00 | Excellent |
| Q4 | 0.016 | 1.00 | Excellent |
| Q5 | 0.016 | 1.00 | Excellent |
| Q6 | 0.313 | 0.27 | Eliminated |
| Q7 | 0.313 | 0.27 | Eliminated |
| Q8 | 0.016 | 1.00 | Excellent |
| Q9 | 0.016 | 1.00 | Excellent |
| Q10 | 0.016 | 1.00 | Excellent |
| Q11 | 0.094 | 0.81 | Excellent |
| Q12 | 0.016 | 1.00 | Excellent |
| Q13 | 0.016 | 1.00 | Excellent |
| Q14 | 0.016 | 1.00 | Excellent |
| Q15 | 0.016 | 1.00 | Excellent |
| Q16 | 0.094 | 0.81 | Excellent |
| Q17 | 0.016 | 1.00 | Excellent |
| Q18 | 0.016 | 1.00 | Excellent |
| Q19 | 0.094 | 0.81 | Excellent |
| Q20 | 0.094 | 0.81 | Excellent |
| Q21 | 0.094 | 0.81 | Excellent |
| Q22 | 0.016 | 1.00 | Excellent |
| Q23 | 0.234 | 0.57 | Eliminated |
| Q24 | 0.094 | 0.81 | Excellent |
| Q25 | 0.234 | 0.57 | Eliminated |
| Q26 | 0.016 | 1.00 | Excellent |
| Q27 | 0.016 | 1.00 | Excellent |
| Q28 | 0.016 | 1.00 | Excellent |
| Q29 | 0.094 | 0.81 | Excellent |
| Q30 | 0.016 | 1.00 | Excellent |
| Q31 | 0.016 | 1.00 | Excellent |
| Q32 | 0.016 | 1.00 | Excellent |
| Q33 | 0.094 | 0.81 | Excellent |
| Q34 | 0.094 | 0.81 | Excellent |
| Q35 | 0.016 | 1.00 | Excellent |

**Table 4: Calculating of CVR for the PEQ (version 5)**

| **Item** | **Scale name/Main content** | **CVR** | **Interpretation** |
| --- | --- | --- | --- |
| Q1 | A supervised exercise program | 1.00 | Remained |
| Q2 | A healthcare provider with a positive attitude toward exercise | 0.00 | Eliminated |
| Q3 | Having friends/family with a positive attitude toward exercise | 0.67 | Eliminated |
| Q4 | An exercise facility in my area | 1.00 | Remained |
| Q5 | Transportation to an exercise facility | 0.67 | Eliminated |
| Q6 | A safe place to exercise | - 0.33 | Eliminated |
| Q7 | An outdoor/indoor area where I can exercise in a supportive and pleasant environment | - 0.33 | Eliminated |
| Q8 | An exercise facility that is free of cost or reasonably priced | 1.00 | Remained |
| Q9 | Have less pain | 1.00 | Remained |
| Q10 | Feel less tired | 1.00 | Remained |
| Q11 | Be able to walk longer | 0.33 | Eliminated |
| Q12 | Be more flexible | 1.00 | Remained |
| Q13 | Have better balance | 1.00 | Remained |
| Q14 | Increase muscle strength | 1.00 | Remained |
| Q15 | Experience less falls | 1.00 | Remained |
| Q16 | Top three exercise goals | - 0.33 | Eliminated |
| Q17 | Exercise pain | 0.66 | Eliminated |
| Q18 | Exercise location | 0.66 | Eliminated |
| Q19 | Exercise group size | 0.00 | Eliminated |
| Q20 | Exercise schedule | 0.33 | Eliminated |
| Q21 | Type of exercise | 0.00 | Eliminated |
| Q22 | Receive feedback about exercise progress | 0.66 | Eliminated |
| Q23 | Type of feedback | 0.00 | Eliminated |
| Q24 | How often would you like to receive feedback about your exercise progress | 0.33 | Eliminated |
| Q25 | Giving feedback on an exercise program | - 0.66 | Eliminated |
| Q26 | Tracking an exercise program | 0.66 | Eliminated |
| Q27 | Do you have things that prevent exercise | 1.00 | Remained |
| Q28 | Exercise fears | 1.00 | Remained |
| Q29 | Exercise difficulties | 0.66 | Eliminated |
| Q30 | Do you think you have barriers to exercise | 1.00 | Remained |
| Q31 | Other medical conditions | 1.00 | Remained |
| Q32 | Additional priorities | 0.00 | Eliminated |
| Q33 | Likelihood of exercising if barriers were limited | 0.66 | Eliminated |
| Q34 | Self-conscious about exercising | 0.33 | Eliminated |
| Q35 | Limited mobility due to fractures | 0.66 | Eliminated |

NOTE: Number of experts evaluated the item essential. CVR = (Ne – N/2)/ (N/2) with 6 person at the expert panel (N = 6), items with the CVR bigger than 0.99 remained in the questionnaire and the rest eliminated.

**Major revisions (in detail)**

The final version of section one, support network, consisted of 3 questions regarding normative beliefs and measured how patients may perceive the attitude of salient individuals and groups toward exercise. Content validity results suggested all three questions were relevant, but questions 2 and 3 (the attitudes of healthcare providers, friends and families toward exercise) were not essential and should be eliminated. Although expert opinions are important to consider there is strong evidence in the literature to support these questions. A systematic review identifying the facilitators and barriers to exercise and a qualitative focus group study on exercise adherence in older adults found physical therapists with a positive attitude toward exercise and a program under the supervision of a physiotherapist of a healthcare professional was a motivator for some patients (19,23). Furthermore, a systematic review of barriers and facilitators to exercise found support from family members and friends, particularly from a spouse, was a positive motivator that encouraged participants to be physically active (41). This study has been corroborated by two literature reviews that identified barriers and facilitators in people with knee OA and older adults (24,42). Questions 2 and 3 were not removed, but rephrased to better capture what the questions were measuring.

Section two had 6 questions in the eighth version and measured how easily participants access an exercise facility. Questions 4 and 8 (exercise facility distance from home/work was and the cost) were considered relevant and essential while items 5 to 7 (transportation, safety, and the type of environment) were marked not essential. In addition, questions 6 and 7 were also marked irrelevant. Although items regarding safety and the type of environment were considered for elimination, qualitative research strongly supports them and they were retained in the tool. Humpel and Owen found a consistent association between environmental factors and exercise behaviours (43) and measuring this concept will be important in understanding the facilitators and barriers to exercise. A study that identified the facilitators and barriers to exercise in people with osteoarthritis reported 52% (12 of the 23) identified factors related to environmental issues (24). Furthermore, a Canadian focus group revealed the following important barriers to exercise: “There is no bus service on the weekend and during the week it is only offered in the early morning or from 4 p.m. to 6 p.m. with limited stops”; “Safe dressing rooms are important. Floors are wet and hooks are too high which means having to stand on benches, this is very dangerous”; “We need instructors that are older, that will not push you like the younger ones, that understand your issues” (21). Qualitative literature strongly supports keeping these questions, however, further research regarding the verification of these environmental components should be considered. It is possible that experts did not understand the questions, since items 5 to 7 were marked unclear. During cognitive interviews with patients and clinicians, these questions were reworded to improve their readability.

The third domain measures exercise goals and patient preferences using 8 questions. In 2003, the World Health Organization (WHO) recommended one method to improve adherence rates was through patient-tailored interventions that take into account patient preferences (44). When patients are not engaged in clinical decision making, they may feel less empowered resulting in lower adherence rates (42). Literature from the Canadian focus group study identified activities of daily living such has being able to lift groceries, climb stairs, play with grandchildren, gardening, walking, and completing housework as important goals. Additional items were also identified from the systematic review that identified the facilitators and barriers to exercise in people with osteopenia and osteoporosis including having less pain and being more flexible (19). Goal setting is an important context to explore and has been found to be a short and a long term barrier to exercise (24). This questionnaire can be used to measure both short and long term goals. Patient reported goals were consolidated into 7 generic objectives. Items like “feeling less tired” encompass many goals such as being able to climb stairs or playing with grandchildren. An open-ended question that asks participants their most important exercise goal was used for dual purpose: (1) to capture other intentions that may have been missed from the previous 7 goal questions; and (2) to force participants to choose one important goal. Cognitive interviews with patients revealed this was the hardest section for most because it required complex, non-algorithmic thinking. Ranking items requires considerable cognitive effort that may cause anxiety. Questions were arranged so that participants required some degree of effort but not to the point of mindlessly answering questions. All items were marked as relevant and essential except question 11 (“be able to walk longer”). This item was also marked unclear, so it’s possible experts did not understand the question. Subsequent versions of the PEQ have improved the clarity of the items in this domain.

Considering a wide range of exercise motives is a novel approach when developing an exercise program (45) and section four identifies possible facilitators to exercise using 7 questions. Experts considered all items relevant but not essential and unclear. After reviewing items with Ph.D. candidates with clinical backgrounds and patients with osteopenia or osteoporosis, questions were found to be biased and/or unclear. Many items in this section originally asked about how exercise reduced pain and were eliminated. All questions were reformed to better represent items that were less biased and focused more on exercise preferences such as preferred exercise locations, exercise group sizes, schedule and type of exercises.

Section five of the PEQ was only included during the third revision by an expert member who suggested the inclusion of this domain since the use of Physical Activity Monitors (PAMs) are becoming a fairly inexpensive and available method to track activity (46). The content validity values for this domain suggested all items should be eliminated although the majority of items were found to be relevant. It is possible that experts believe that many older adults track exercise habits using the pencil and paper method or their memory and that this domain was unnecessary. However, there is evidence supporting older adults are becoming more interested in and capable in using technology to track exercise behaviours (46,47). There is strong evidence that tacking ones exercise habits and receiving positive reinforcement improves exercise adherence (48). Although experts believe this domain to be redundant previous evidence has shown tracking increases exercise behaviours and understand how patients would like to track their progress may be important. Pilot testing of this instrument may determine the usefulness of this section.

Barriers to exercise may be considered one of the strongest influencers that prevent people from exercising. Section six may be considered the most important part of the questionnaire and consists of 8 questions identified through qualitative research in the osteoarthritis population and women’s focus group study from Canada (21,42). Additional items were identified from a systematic review that looked at facilitators and barriers in the general population (41). Barriers identified included inconvenient class sessions, cost and location, intimidating gym atmosphere, dislike of the music and/or television and lack of confidence when operating gym equipment. All items were considered relevant, however most were marked not essential and unclear. Considering all questions were marked as relevant, it is possible that the clarity of items resulted in them being ranked not essential. Feedback from clinicians and patients helped design clearer and more succinct questions. Barriers may be more important than facilitators since this may affect adherence more than other factors. For example a person with accesses to an exercise facility may be impeded due to financial costs. In some cases barriers may outweigh all factors and investigators should weight barriers to exercise more strongly than facilitators.

The overall layout of the tool was designed to minimize boredom and combines cognitively difficult questions with easy ones. Stone et al recommends the initial part of a question be neutral but interesting with more sensitive items at the end (26). This questionnaire was designed get progressively more difficult with more challenging questions followed by some easier items to alleviate cognitive load. Section one and two were chosen as the first and second domain since they are relatively easy to complete. The third section is also simple, but requires a little rational since it asks participants to rank their exercise goals. Section four, starts of with an open-ended question that may require some time to complete, but is then followed by easier items. An easier section regarding tracking and feedback during an exercise program follows section four. The last section, barriers to exercise, requires the most cognitive skills and takes the most time. This questionnaire takes about 20 to 30 minutes to complete.

A 4-point Likert scale was used in domains 1, 2 and 3. The reliability of a 5 to 7 point Likert scale is stronger than a 4-point scale, however the 4-point scale was more appropriate since most patients with osteoporosis are 50 plus and smaller scales are simple to use and less cognitively demanding. A 4-point ordinal scale also avoids having neutral or ambivalent midpoint answers. Lastly, unlike other measures that are completed at multiple time points, respondents complete this questionnaire once and it is unnecessary to test the accuracy of answer choices.

There are a couple of questions in this instrument that may seem redundant and have overlapping themes. For example question 6 in domain 2 assesses participants transportation to an exercise facility, similar to question 34 in section 6. These repeat questions act as decoys to determine mismatches in participant answers. Possible conflicting answers may indicate item misinterpretation.

The PEQ was designed to identify possible facilitators and barriers to exercise in patients with osteopenia and osteoporosis. A series of questions have been posed in a clear, comprehensible, and appropriate manner so that respondents can for formulate, articulate and transmit their answers effectively. Although this questionnaire was intended for people with osteoporosis, it may be suited for older adults since many items were identified from literature in mature adults.
