# Supplementary material for: Development and validation of a new tool to measure the facilitators, barriers and preferences to exercise in people with osteoporosis
Source: BMC Musculoskelet Disord. 2017 Dec 19;18:540. doi: 10.1186/s12891-017-1914-5 (PMC5738121; doi:10.1186/s12891-017-1914-5)
Supplement: Supplementary file 3 — Appendix C. The Personalized Exercise Questionnaire (PEQ). The PEQ was designed to identify possible facilitators and barriers to exercise in patients with osteopenia and osteoporosis. There are 6 domains (support network, access, goals, preferences, feedback and tracking, and barriers) and 38 questions. (PDF 121 kb) [file 12891_2017_1914_MOESM3_ESM.pdf]

ID: ______________________________

Personalized Exercise Questionnaire (PEQ)

PLEASE READ THESE INSTRUCTIONS BEFORE STARTING:

This survey was created to better understand your **exercise needs** and **goals**. By completing this survey you will help us understand some of the difficulties you face in an exercise program. This information will be used to help us create better exercise/ physical activity program for you.

There are **6 sections** and **38 questions**. Please complete **ALL** questions relevant to you. **All answers will be kept strictly confidential and never associated with your name**

SECTION ONE: My Support Network

	No	Not Sure	Yes	Not Applicable
1. I prefer someone to supervise/ assist me with an exercise:	☐	☐	☐	☐
2. A healthcare provider (e.g. physiotherapist, nurse, etc.) with a good attitude toward exercise is important to me:	☐	☐	☐	☐
3. Having friends/family with a good attitude toward exercise is important to me:	☐	☐	☐	☐

If **YES**, under a (question 1):

- ☐ Healthcare professional (e.g. physiotherapist)
- ☐ Personal Trainer
- ☐ Other: ________________

ID: ______________________________

SECTION TWO: My Access to Exercise

	No	Not Sure	Yes	Not Applicable
4. I have a place to exercise (indoor or outdoor) at home, place of work or near my home/work place:	☐	☐	☐	☐

If **YES**, how far:

- ☐ At home or at work
- ☐ < 5 km (< 3 miles)
- ☐ 5 – 10 km (3-6 miles)

	No	Not Sure	Yes	Not Applicable
5. I am able to get to an exercise site on my own: (Check "Not Applicable" if you exercise at home)	☐	☐	☐	☐

If **NO**, who could you ask:

- ☐ Family member/partner
- ☐ Friend
- ☐ Other: ______________

	No	Not Sure	Yes	Not Applicable
6. I have transportation to an exercise site: (Check "Not Applicable" if you exercise at home)	☐	☐	☐	☐

If **YES**, type of transportation:

- ☐ Bike
- ☐ Motor Vehicle (e.g. car)
- ☐ Public transportation
- ☐ Walking

	No	Not Sure	Yes	Not Applicable
7. I have a safe place to exercise: (e.g. proper space to exercise, dry and clean floors, good lighting, etc.	☐	☐	☐	☐
8. I have an encouraging place to exercise: (e.g. pleasant people that motivate me)	☐	☐	☐	☐

ID: ______________________________

	No	Not Sure	Yes	Not Applicable
9. I have an exercise location that is free of cost or reasonably priced (including parking fees):	☐	☐	☐	☐

SECTION THREE: My Exercise Goals

How important are the following GOALS to YOU in an exercise program?

	Not Important	Somewhat Important	Very Important	Not Applicable
10. Feel less tired	☐	☐	☐	☐
11. Be able to walk longer	☐	☐	☐	☐
12. Be more flexible	☐	☐	☐	☐
13. Have better balance	☐	☐	☐	☐
14. Fall less often	☐	☐	☐	☐
15. Have less pain	☐	☐	☐	☐
16. Increase muscle strength	☐	☐	☐	☐

17. What is your MOST important exercise goal?

____

SECTION FOUR: My Exercise Preferences

18. Please list up to 3 things that HELP you to exercise more often:

1. ____
2. ____
3. ____

ID: ______________________________

19. Where would you like your exercise program to be? (Check **ALL** that apply)

- ☐ Home
- ☐ Gym (e.g. YMCA/YWCA, Goodlife Fitness, Wynn Fitness, etc.)
- ☐ Mall
- ☐ Community Centre
- ☐ Outdoors (e.g. parks, trails, sidewalks, etc.)
- ☐ Other: ________________________________

20. What is the best time for you to exercise? (Check **ALL** that apply)

- ☐ Morning (between 6:00 am to 12:00 pm)
- ☐ Afternoon (between 12:00 pm to 6:00 pm)
- ☐ Evening (between 6:00 pm to 11:00 pm)

21. What is your preferred exercise schedule? (Check **ALL** that apply)

- ☐ Fixed time (same class offered at same time during the week)
- ☐ Multiple drop-in times (same class offered at different times of the week)
- ☐ On my own time

22. What is your preferred exercise class size? (Check **ALL** that apply)

- ☐ I prefer to exercise alone
- ☐ With a partner/trainer
- ☐ Small group (less than 10 people)
- ☐ Large group (more than 10 people)
- ☐ Does not matter

23. How would you like to learn proper exercise technique? (Check ALL that apply)

- ☐ Taught by a healthcare professional (e.g. physiotherapist, nurse, etc.)
- ☐ Taught by a trainer/health club staff
- ☐ Learn on my own from an exercise video
- ☐ Learn on my own from a website with pictures
- ☐ Learn on my own using an app
- ☐ Learn on my own using a print handout
- ☐ Have a friend teach me
- ☐ Have another person with osteoporosis teach me
- ☐ None of the above

ID: ______________________________

24. What level of exercise are you comfortable doing? (Check **ALL** that apply)

- ☐ Easy to perform
- ☐ Challenging to perform (i.e. "I like a challenge")
- ☐ Slow paced exercises
- ☐ Fast paced exercises
- ☐ Easy to remember

SECTION FIVE: My Feedback and Tracking

25. I would like to receive feedback about my progress:

- ☐ YES
- ☐ NO

If **YES**, by: (Check **ALL** that apply)

- ☐ Email
- ☐ In person
- ☐ Social media (e.g. Twitter, Facebook, etc.)
- ☐ Phone call
- ☐ Text message

*If you answered **YES** to question 25, please complete questions 26 and 27.*

*If you answered **NO** to question 25, please skip to question 28.*

26. What type of feedback would you like to receive? (Check **ALL** that apply)

- ☐ Regarding my exercise progress and future improvements
- ☐ Regarding proper exercise techniques
- ☐ Other: ______________________________

27. How often would you like to receive feedback about your exercise progress? (Please check only **ONE** answer)

- ☐ Daily
- ☐ Weekly
- ☐ Monthly
- ☐ Yearly

ID: ________________________________

28. I would like to give feedback on the exercise program:

☐ YES
☐ NO

If **YES**, by (Check **ALL** that apply):

☐ Email
☐ In person
☐ Social media (e.g. Twitter, Facebook, etc.)
☐ Phone call
☐ Text message

29. I would like to track my exercise progress:

☐ YES
☐ NO

If **YES**, using (Check **ALL** that apply):

☐ Cellphone/mobile
☐ Diary/Log book
☐ Wearable technology (e.g. Fit Bit, pedometer, watch etc.)
☐ Other: ______________________________________

SECTION SIX: My Barriers to Exercise

30. Do you have things that STOP you from exercising?

☐ Yes
☐ No

If **YES**, how often does it stop you from exercising: (Check only **ONE** answer)

☐ Always
☐ Very often
☐ Sometimes
☐ Rarely

31. Please list up to 3 things that STOP you from exercising more often:

1. ____

2. ____

3. ____

ID: ______________________________

32. I do not exercise as often as I like because: (Check **ALL** that apply)

- ☐ I do not like exercise
- ☐ I do not want to fall
- ☐ I do not want to injure myself (e.g. breaking a bone or bruising)
- ☐ I feel pain when I exercise
- ☐ I feel bored when exercising
- ☐ Other: ____
- ☐ None of the above

33. I do not exercise as often as I like because I have difficulty: (Check **ALL** that apply)

- ☐ Understanding the exercise
- ☐ Performing the exercise (i.e. I do not know how to exercise safely)
- ☐ Other: ____
- ☐ None of the above

34. I do not exercise as often as I like because I do not have: (Check **ALL** that apply)

- ☐ A place to exercise
- ☐ Confidence (e.g. I feel self-conscious about my body)
- ☐ Finances
- ☐ Mobility (e.g. limited movements due to pain)
- ☐ Proper quality of sleep
- ☐ Transportation
- ☐ Time (e.g. family priorities, work, etc.)
- ☐ Willpower/motivation
- ☐ Other: ____
- ☐ None of the above

35. Do weather conditions stop you from exercising as often as you like? (Check only **ONE** answer)

- ☐ Always
- ☐ Very often
- ☐ Sometimes
- ☐ Rarely
- ☐ Never

ID: ________________________________

36. I do not exercise as often as I like because I have medical conditions such as: (Check **ALL** that apply)

- ☐ Arthritis (e.g. hips, knees, etc.)
- ☐ Cognitive concerns (e.g. Alzheimer, Dementia, Parkinson, etc.)
- ☐ Heart condition (e.g. angina, heart failure, etc.)
- ☐ Kidney disease (e.g. dialysis)
- ☐ Lung disease (e.g. asthma, COPD, etc.)
- ☐ Mental health issues (e.g. anxiety, depression, etc.)
- ☐ Other: ____
- ☐ None of the above

37. If you had fewer barriers would you spend more time exercising? (Please check only **ONE** answer)

- ☐ Yes
- ☐ No
- ☐ Not sure

38. Please check any mobility aids that you normally use:

- ☐ Cane
- ☐ Walker
- ☐ Crutches
- ☐ Wheelchair
- ☐ Other: ____
- ☐ None

☺ *End* ☺
